# The association between emergency department length of stay and hospital length of stay: an observational multi-centre cohort study

**DOI:** 10.1007/s11739-025-03964-w

**Published:** 2025-05-26

**Authors:** Merel van Dijk, Menno I. Gaakeer, Marianne Jonker, David N. Baden, Bas de Groot

**Affiliations:** 1 Department of Emergency Medicine, Radboudumc, Geert Groote Plein Zuid 22, 6525 GA Nijmegen, The Netherlands; 2 Department of Emergency Medicine, Adrz Hospital, ‘s-Gravenpolderseweg 114, 4462 RA Goes, The Netherlands; 3 Department of Health Evidence, Biostatistics Research Group, Radboudumc, Geert Grooteplein 21, 6525 EZ Nijmegen, The Netherlands; 4 Department of Emergency Medicine, Diakonessenhuis, Burgemeester Fockema Andreaelaan 60, 3582 KD Utrecht, The Netherlands; 5 Aarhus University Hospital, Research Center for Emergency Medicine, Department of Clinical Medicine, Palle Juul-Jensens Boulevard 99, J103, 8200 Aarhus N, Denmark

**Keywords:** Emergency department length of stay, Crowding, Emergency medical services, Hospital length of stay

## Abstract

Prolonged emergency department (ED) length of stay (LOS) is associated with ED crowding which is linked to mortality in healthcare systems with relatively long ED LOS. We hypothesized that even in systems with shorter ED LOS, prolonged ED LOS is also associated with increased hospital LOS, particularly in older and urgently triaged patients. This study assesses the association between ED LOS and hospital LOS ≥ 3 days in patients older and younger than 70 years, categorized by triage urgency. Observational multicentre cohort study including all hospitalized ED patients from the Netherlands Emergency department Evaluation Database (NEED), stratified by age and triage category. The NEED contains data from seven EDs of two tertiary care centres and four urban hospitals. Multivariable logistic regression analyses were employed to calculate Adjusted Odds Ratios (AOR) for the association between ED LOS and hospital LOS ≥ 3 days, adjusting for confounders. Of the 718,358 patients 258,636 adults were hospitalized. Median ED LOS of hospitalized patients was 3.30 h (95% CI 3.30–3.31), while median hospital LOS was 3.0 (95% CI 2.98–3.02) days; 12,511 patients (2%) died. Patients with ED LOS of 4–8 and > 8 h had AORs for hospital LOS ≥ 3 days of 1.39 (95% CI 1.36–1.41) and 1.58 (95% CI 1.50–1.66), respectively, compared to patients with ED LOS < 4 h. In the Dutch healthcare system, which has a relatively short ED LOS, prolonged ED LOS is associated with an increased hospital LOS. This association appears to be more pronounced in patients who are younger and triaged less urgent.

**Supplementary Information:**

The online version contains supplementary material available at 10.1007/s11739-025-03964-w.

## Introduction

Prolonged emergency department (ED) length of stay (LOS) is associated with in-hospital mortality in healthcare systems in which ED LOS is relatively long [1–6]. Prolonged ED LOS is associated with ED overcrowding which is a situation that occurs when the identified need for emergency services exceeds available resources for patient care in the ED, hospital, or both [7]. This leads to effective capacity decrease and has been associated with worse patient outcomes and satisfaction among health workers and patients [1, 8–11]. Crowding is also a problem in the Netherlands; 68% of the ED managers experienced crowding several times a week or even daily [12, 13]. Besides mortality, it is important to consider the impact of ED LOS on prolonged hospital LOS as this is related to costs [14], but have not been studied extensively before. In addition, it is unclear whether ED LOS is associated with increased hospital LOS in healthcare systems with relatively short ED LOS, such as the Netherlands. Although debated, recent extrapolations by the Dutch Society of Emergency Physicians of findings of an English study suggested approximately 900 avoidable deaths per year [6, 15, 16] in Dutch EDs. If in the Netherlands, prolonged ED LOS is also linked to increased hospital LOS, it would be relevant to combat ED crowding by reducing ED LOS, not only for safety reasons but also for economic reasons.

Importantly, the association between ED LOS and prolonged hospital LOS might differ based on patients’ age and triage category. Older patients, due to reduced physiological reserve, more co-morbidity, and increased susceptibility to delirium, might be more sensitive to treatment delays. Similarly, patients with more severe illnesses could also be significantly impacted by prolonged ED LOS. For example, ED LOS may not be associated with prolonged hospital LOS in ED patients with an ankle distortion (categorized as non-urgent in triage systems), while hospital LOS may be longer in ED patients with septic shock (categorized as very urgent in triage systems) who have delays in their treatment or are not transferred timely to an appropriate level of care [17].

Therefore, the objectives of this study were (1) to assess the association between ED LOS and hospital LOS, and (2) to explore whether this association is affected by age and disease severity. We hypothesized that an ED LOS longer than 4 h is associated with longer hospital LOS in the older and sicker patients, but not in younger patients with lower triage urgency.

## Methods

### Study design and setting

**Fig. 1:**
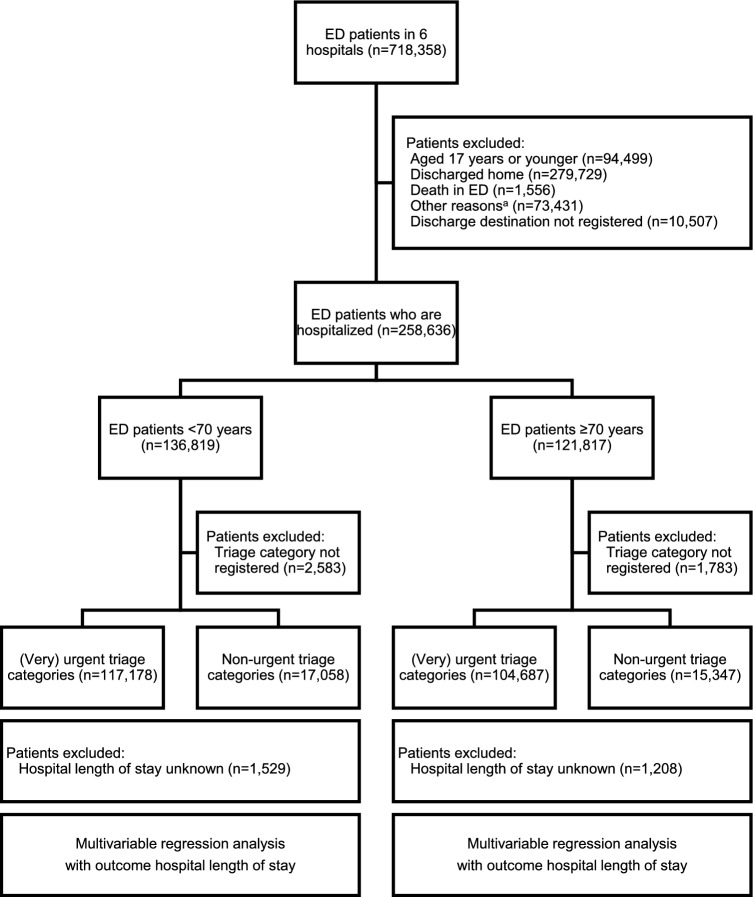
Patient inclusion and flow through study. Disease severity was indicated by triage category (non-urgent: green/blue, urgent: yellow/orange/red). ^a^Other reasons: discharge against medical advice, left without seeing doctor, self-initiated departure, left before completion of treatment, transfer to another hospital, scheduled outpatient check-up or out-of-hours GP service. *ED* emergency department; *GP* general practitioner

This was an observational multicentre cohort study in which patients were stratified by age (≥ 70 and < 70 years) and triage urgency (non-urgent (blue and green) and (very) urgent (yellow, orange and red); see Fig. 1). We used data from the Netherlands Emergency department Evaluation Database (NEED). The NEED is the Dutch quality registry of Eds and contained data from seven Eds in six Dutch hospitals: two tertiary care centres with an annual census of 16,000–20,000 ED visits, and 4 urban hospitals with approximately 20,000–36,000 ED visits annually, typical numbers for Dutch ED’s. The two of the seven included tertiary care centres in the Netherlands are University Medical Centres and level 1 trauma centres and have all facilities (Thoracic and neuro surgery, Percutaneous Coronary Intervention, Intra Arterial Thrombectomy, ICUs, etc.). One is located in the eastern part of the Netherlands and one is located in the western part of the Netherlands. The four teaching hospitals are located in different regions in the Netherlands. They have a varying range of facilities but not all facilities. Together they are a representative sample of the Dutch ED landscape. In general, work flows in the Netherlands are aimed to finish ED work-up within 4 h. Since blood tests, radiological testing (especially CT scans and ultrasounds) and consultations of other specialties account for most of the time spend in the ED [18], it is attempted to initiate this as early as possible. In many Dutch EDs, blood tests are taken by nurses by the time of triage (advance triage). In some hospitals, there are extra radiologists in training present in the ED during busy hours (evening shifts) or an acute radiologist is present in the ED. In many hospitals, patients from the ED are initially admitted to an Acute Admission Ward. In most participating EDs of this study, there are 24/7 emergency physicians. One study showed that from 63 of 94 EDs in the Netherlands in 2012, the mean ED LOS of patients discharged from the ED was 119 (SD 40) min. while the mean ED LOS of patients admitted to the hospital was 146 (SD 49) min [13].

The medical ethics review committee of the Radboudumc (METC Oost) declared that the research did not fall under the Medical Research Act, and waived the need for informed consent since this was an observational study (file no. 2023-16299).

### Study population

#### Inclusion criteria

Consecutive ED patients who visited the ED between 1 January 2017 and 31 December 2022 were included.

#### Exclusion criteria

Patients aged 17 years or younger.

#### Data collection

From the participating hospitals and included patients, information is collected through the NEED about patient, ED and hospital characteristics. Detailed information about the information acquired in the NEED is available in Supplemental File 1.

#### Study parameters

Detailed information about study parameters is available in Supplemental File 1.

Briefly, the following variables were collected: Patient characteristics: Demographics (age, gender), triage category and presenting complaints, vital signs, time and method of referral and arrival, type and number of diagnostic tests. As a proxy for comorbidity and complexity of patients we used the number of consultations in the ED and performance of laboratory or radiological tests [18, 19]. ED length of stay has been calculation by subtraction of the time of ED registration at the ED registration desk from the time of ED discharge.

### Study outcomes

The primary outcome was hospital LOS equal to or exceeding the median LOS. Patients who were brought into the ED already deceased (which may occur in cases requiring cardiopulmonary resuscitation) or who died while in the ED were excluded, primarily based on the rationale that the ED LOS was unlikely to have exerted any influence on their outcome.

### Sample size calculation

We used the rule of thumb that approximately 5–10 events per variable are needed to prevent overfitting in association models [20, 21]. We aimed to adjust for ~ 23 potential confounders in the regression analysis with hospital LOS ≥ 3 days as outcome. We therefore needed approximately 230 events per age/disease severity category to prevent overfitting. On average, we have 0.33 × 700,000 = 233,333 hospitalized patients [15, 22]. Of whom approximately 116,500 will be hospitalized for more than median hospitalization duration of 3 days. We expected, therefore, to have more than enough events per variable to adjust for the planned 23 potential confounders.

### Statistical analysis

#### Descriptive statistics

Data were presented as mean (standard deviation (SD)) when normally distributed. Skewed data were presented as median (interquartile range (IQR)). Categorical data were presented as number (%).

Differences among strata with respect to outcomes were assessed as follows: Continuous data were compared using student t-tests or Mann–Whitney U-tests, as appropriate. Descriptive categorical data were analyzed using chi-square tests.

#### Objective 1 and 2: main statistical analyses

In Fig. 1, a schematic representation of the study design and analysis plan is shown. We considered age and triage urgency to be effect modifiers of the primary associations of interest. Our hypothesis was that the association between ED LOS and hospital LOS would be different in ED patients aged 70 years or older compared to ED patients younger than 70 years, and in ED patients with low and high triage urgence. For this reason, we developed multivariable regression models for hospital LOS in the two age categories (< and ≥ 70 years) and the two triage categories (low and high triage urgency). Because we considered that the primary determinant ED LOS might not to be linearly associated with the primary outcomes, we categorized ED LOS in categories < 4 h, 4–8 h, and > 8 h. The 4 h cut-off was chosen because in the Netherlands (but also other countries) 4 h is often considered a time span in which ED work-up, i.e. taking history, physical examination, diagnostic testing and decision making, can be finished. To assess whether ED LOS was associated with the primary outcome, we developed association models [20, 21] using multivariable logistic regression analyses with backward stepwise elimination of variables with *P* > 0.05. In the multivariable regression analyses, the following potential confounders were included: patient demographics (age and sex), presenting complaint (most frequent top 10), vital signs (measured or not) Glasgow coma scale (GCS), number of consultations in the ED, laboratory and radiology testing ED (0 = no, 1 = yes) and discharge destination (ward/MCU/ICU; as a measure of disease severity). Because comorbidities are not registered in the NEED, we used proxies (consultations, laboratory and radiology testing) of co-morbidities and complexity as it was previously shown that these proxies are associated with comorbidities and complexity [18, 19].

Unadjusted and adjusted Odds Ratios (OR) and other outcomes were reported with 95% confidence intervals (95% CI). A *P* value < 0.05 was considered to be statistically significant. We corrected for multiple comparisons according to the method of Bonferroni. *P* values were therefore multiplied by 3 as there are 4 age/disease severity groups).

Data were analyzed using SPSS (SPSS, version 29.0, IBM, New York, USA).

We have added analyses without dichotomization, i.e. with predictors as continuous or ordinal variables. We used the R package mgcv15, to fit a generalized additive logistic regression model (GAM) to the binary outcome hospital LOS > 3 days. In this model, the log odds of the outcome depends in an arbitrary way on all predictors (ED-LOS, age and triage category) and all their interactions. Clearly, such a complex model is overparameterized which could lead to poor, unstable performance if left unaddressed. However, it can be handled as part of the fitting procedure by using a quadratically penalized likelihood type approach. Effectively, this method enforces a smooth dependence of the log odds mortality on the three predictors. To visualize the association between the two outcomes and the predictors, we generated a graph. The stratified analyses using multivariable logistic regression analyses allowed us to adjust for multiple confounders, while the GLM allowed us to verify that the results were similar and not changed by the stratification/dichotomization.

#### Sensitivity analyses

To investigate the impact of the way in which we adjust for disease severity we used various other proxies of disease severity like triage category, GCS, additional testing, and discharge destination in all regression models. In addition, we did a sensitivity analysis without ICU patients. Finally, we tested whether the results were affected by the COVID period by leaving out the patients who visited the ED between 11 March 2020 and 31 December 2022.

## Results

### Patient inclusion and characteristics

In Fig. 1, patient flow through study is shown. Of the 718,358 patients, 258,899 adult patients were hospitalized. Of the hospitalized patients, the majority was younger than 70 years and triaged as urgent. The median ED LOS for the entire cohort was 2.60 h (2.59–2.61).

Table 1 shows patient characteristics of all the hospitalized patients and stratified in patients who were hospitalized < 3 or ≥ 3 days. Of the hospitalized ED patients (average age: 66 years, 53% male), 44% presented between 12:00 and 17:59. The most frequent presenting complaints were feeling unwell (25%), shortness of breath (14%), and abdominal pain (12%). Patients who were hospitalized ≥ 3 days were more often transported by ambulance than younger patients. In addition, they were more often triaged as urgent, and were more often treated with oxygen, fluids and medication. Patient characteristics of all 718,358 patients who visited the ED can be also found in Supplemental File 2. Hospitalized patients were generally older, more frequently arrived by ambulance, and presented more often with non-specific complaints, and were triaged more often as (very) urgent compared to the overall cohort. In Table 2, patient characteristics are stratified by ED LOS < 4, 4–8, and > 8 h. Disease severity, complexity and resource use appear to be higher in patients with ED-LOS > 8 h.

**Table 1:** Characteristics of patients who were admitted through the emergency department stratified into patients with a hospital lengths of stay more or less than 3 days

	Total cohort (*n = *255,899)	Hospital LOS < 3 days (*n = *99,255)	Hospital LOS ≥ 3 days (*n = *156,644)
Demographics			
Age, mean (SD)	66 (17)	61 (19)	69 (16)
Male sex, *n* (%)	135,435 (53)	52,686 (53)	82,749 (53)
Previous ED attendances, median (IQR)	2 (3)	2 (2)	2 (3)
Patient pathway, *n* (%)			
Type of hospital			
Academic	49,717 (19)	18,507 (19)	31,210 (20)
Urban	206,182 (81)	80,748 (81)	125,434 (80)
Type of arrival			
Private transport	112,841 (44)	47,330 (48)	65,511 (42)
Ambulance	125,610 (49)	45,746 (46)	79,864 (51)
Not registered	17,448 (7)	6179 (6)	11,269 (7)
Referral source			
Self-referral	79,351 (31)	34,820 (35)	44,531 (28)
GP	140,713 (55)	51,620 (52)	89,093 (57)
Specialist	29,501 (12)	9910 (10)	19,591 (13)
Not registered	6334 (2)	2905 (3)	3429 (2)
Top 10 presenting complaints, *n* (%)			
Feeling unwell	64,455 (25)	19,446 (20)	45,009 (29)
Shortness of breath	36,476 (14)	8451 (9)	28,025 (18)
Abdominal pain	30,583 (12)	13,877 (14)	16,706 (11)
Chest pain	19,585 (8)	10,887 (11)	8698 (6)
Extremity complaints	16,680 (7)	5208 (5)	11,472 (7)
Trauma, severe	9567 (4)	4326 (4)	5241 (3)
Collapse	5672 (2)	2907 (3)	2765 (2)
Vomiting, diarrhea	4481 (2)	1583 (2)	2898 (2)
Fallen	3713 (2)	1216 (1)	2497 (2)
Wounds	3189 (1)	1711 (2)	1478 (1)
Triage category, *n* (%)			
Green/blue	31,711 (12)	13,457 (14)	18,254 (12)
Yellow	134,393 (53)	51,983 (52)	82,410 (53)
Orange	75,224 (29)	27,944 (28)	47,280 (30)
Red	10,301 (4)	4153 (4)	6148 (4)
Not registered	4270 (2)	1718 (2)	2552 (2)
Registered medical specialty*n* (%)			
ED physician	16,066 (6)	6436 (7)	9630 (6)
Surgical specialty	52,294 (20)	22,459 (23)	29,835 (19)
Medical specialty	187,491 (73)	70,334 (71)	117,157 (75)
Not registered	48 (0)	26 (0)	22 (0)
Number of consultations, *n* (%)			
0	132,927 (52)	52,206 (53)	80,721 (52)
1	89,417 (35)	35,722 (26)	53,695 (34)
2	27,892 (11)	9454 (10)	18,438 (12)
≥ 3	5619 (2)	1851 (2)	3768 (2)
Not registered	44 (0)	22 (0)	22 (0)
Vital signs at ED presentation			
Registered vital signs, *n* (%)			
None registered	14,300 (6)	7,260 (7)	7,040 (5)
A few registered	68,069 (27)	28,280 (29)	39,789 (25)
All registered	173,540 (68)	63,715 (64)	109,815 (70)
Respiratory rate (per minute), median (IQR)	18 (6)	16 (6)	18 (7)
Oxygen saturation (%), median (IQR)	97 (4)	98 (3)	96 (4)
Heart rate (per minute), mean (SD)	89 (22)	86 (22)	90 (22)
Systolic BP (mmHg), mean (SD)	141 (28)	142 (27)	140 (28)
Diastolic BP (mmHg), mean (SD)	81 (18)	83 (17)	80 (18)
GCS, *n* (%)			
Not registered	179,196 (70)	69,410 (70)	109,786 (70)
Registered, < 15	8966 (4)	3331 (3)	5635 (4)
Registered, = 15	67,737 (27)	26,514 (27)	41,223 (26)
NRS Pain score, *n* (%)			
Not registered or 0	189,439 (74)	72,239 (73)	117,200 (75)
1–3	30,240 (12)	12,190 (12)	18,050 (12)
4–6	22,572 (9)	9136 (9)	13,436 (9)
7 +	13,648 (5)	5690 (6)	7958 (5)
Temperature (°C), mean (SD)	37.0 (1.0)	36.8 (0.9)	37.1 (1.1)
Diagnostic testing, *n* (%)			
Laboratory tests	237,076 (93)	87,285 (88)	149,791 (96)
ECG	135,309 (63)	44,236 (45)	91,073 (58)
Radiological examination	180,843 (71)	61,722 (62)	119,121 (76)
Treatment information, *n* (%)			
Oxygen administered	55,555 (22)	17,313 (17)	38,242 (24)
Fluids administered			
None	193,873 (76)	81,194 (82)	112,679 (72)
≤ 500 ml	35,668 (14)	11,039 (11)	24,629 (16)
> 500 ml	26,358 (10)	7022 (7)	19,336 (12)
Medication administered	117,737 (46)	39,582 (40)	78,155 (50)

Data are presented as mean (SD) if normally distributed or as median (IQR) if skewed. Categorical data are presented as number (%)

*BP* blood pressure; *ED* emergency department; *ECG* electrocardiogram; *GCS* Glasgow Coma Scale; *GP* general practitioner; *IQR* interquartile range; *LOS* Lengths of Stay, NRS, numeric rating scale; *SD* standard deviation

**Table 2:** Characteristics of patients who were admitted through the emergency department stratified into emergency department length of stay

	Total cohort (*n = *258,285)	ED LOS < 4 h (*n = *167,198)	ED LOS 4–8 h (*n = *83,624)	ED LOS > 8 h (*n = *7,463)	*P*
Demographics					
Age, mean (SD)	66 (17)	65 (18)	66 (17)	68 (17)	< 0.001
Male sex, *n* (%)	136,707 (53)	89,555 (54)	43,310 (52)	3842 (52)	< 0.00 1
Previous ED attendances, median (IQR)	2 (3)	2 (3)	2 (3)	2 (3)	< 0.001
Patient pathway, *n* (%)					
Type of hospital					< 0.001
Academic	49,810 (19)	26,565 (16)	21,059 (25)	2186 (29)	
Urban	208,475 (81)	140,633 (84)	62,565 (75)	5277 (71)	
Type of arrival					< 0.001
Private transport	114,115 (44)	70,592 (42)	40,323 (48)	3200 (43)	
Ambulance	126,669 (49)	84,196 (50)	38,376 (46)	4097 (55)	
Not registered	17,501 (7)	12,410 (7)	4925 (6)	166 (2)	
Referral source					< 0.001
Self-referral	80,095 (31)	54,960 (33)	22,818 (27)	2,317 (31)	
GP	142,160 (55)	90,984 (54)	47,130 (56)	4046 (54)	
Specialist	29,617 (11)	16,742 (10)	11,884 (14)	991 (13)	
Not registered	6413 (2)	4512 (3)	1792 (2)	109 (1)	
Top 10 presenting complaints, *n* (%)					< 0.001
Feeling unwell	64,788 (25)	38,173 (23)	24,430 (29)	2185 (29)	
Shortness of breath	36,653 (14)	22,932 (14)	12,656 (15)	1065 (14)	
Abdominal pain	30,943 (12)	18,230 (11)	11,583 (14)	1130 (15)	
Chest pain	19,659 (8)	15,515 (9)	3745 (5)	399 (5)	
Extremity complaints	17,007 (7)	11,396 (7)	5285 (6)	326 (4)	
Trauma, severe	9630 (4)	6236 (4)	3117 (4)	277 (4)	
Collapse	5706 (2)	3749 (2)	1760 (2)	197 (3)	
Vomiting, diarrhea	4496 (2)	2764 (2)	1630 (2)	102 (1)	
Fallen	3876 (2)	2454 (2)	1187 (1)	235 (3)	
Wounds	3282 (1)	2539 (2)	700 (1)	43 (1)	
Triage category, *n* (%)					< 0.001
Green/blue	32,375 (13)	21,172 (13)	10,671 (13)	532 (7)	
Yellow	135,570 (53)	83,104 (50)	48,050 (57)	4416 (59)	
Orange	75,677 (29)	50,498 (30)	22,809 (27)	2370 (32)	
Red	10,356 (4)	8,765 (5)	1,475 (2)	116 (2)	
Not registered	4307 (2)	3659 (2)	619 (0)	29 (0)	
Registered medical specialty, *n* (%)					< 0.001
ED physician	16,339 (6)	8698 (5)	7043 (8)	598 (8)	
Surgical specialty	53,091 (21)	35,417 (21)	16,010 (19)	1664 (22)	
Medical specialty	188,803 (73)	123,042 (74)	60,561 (72)	5200 (70)	
Not registered	52 (0)	41 (0)	10 (0)	1 (0)	
Number of consultations, *n* (%)					< 0.001
0	134,596 (52)	92,290 (55)	38,940 (47)	3366 (45)	
1	90,077 (35)	59,803 (36)	28,313 (34)	1961 (26)	
2	27,955 (11)	12,961 (8)	13,517 (16)	1477 (20)	
≥ 3	5608 (2)	2106 (1)	2844 (3)	658 (9)	
Not registered	49 (0)	38 (0)	10 (0)	1 (0)	
Vital signs at ED presentation					< 0.001
Registered vital signs, *n* (%)					
None registered	14,643 (6)	11,504 (7)	2969 (4)	170 (2)	
A few registered	68,671 (27)	45,757 (27)	21,645 (26)	1,269 (17)	
All registered	174,971 (68)	109,937 (66)	59,010 (71)	6024 (81)	
Respiratory rate (per minute), median (IQR)	18 (6)	17 (6)	18 (6)	18 (7)	< 0.001
Oxygen saturation (%), median (IQR)	97 (4)	97 (4)	97 (4)	96 (4)	< 0.001
Heart rate (per minute), mean (SD)	89 (22)	88 (22)	90 (21)	90 (22)	< 0.001
Systolic BP (mmHg), mean (SD)	141 (28)	142 (28)	139 (28)	140 (28)	< 0.001
Diastolic BP (mmHg), mean (SD)	81 (18)	82 (17)	80 (18)	80 (18)	< 0.001
GCS, *n* (%)					< 0.001
Not registered	181,007 (70)	116,460 (70)	59,675 (71)	4872 (65)	
Registered, < 15	8996 (4)	6001 (4)	2619 (3)	376 (5)	
Registered, = 15	68,282 (26)	44,737 (27)	21,330 (26)	2,215 (30)	
NRS Pain score, *n* (%)					< 0.001
Not registered or 0	190,663 (74)	124,270 (74)	61,488 (74)	4,905 (66)	
1–3	30,789 (12)	20,906 (13)	8882 (11)	1001 (13)	
4–6	22,983 (9)	14,098 (8)	7977 (10)	908 (12)	
7 +	13,850 (5)	7924 (5)	5277 (6)	649 (9)	
Temperature (°C), mean (SD)	37.0 (1.0)	36.9 (1.0)	37.1 (1.1)	37.1 (1.1)	< 0.001
Diagnostic testing, *n* (%)					
Laboratory tests	238,990 (93)	151,530 (91)	80,222 (96)	7238 (97)	< 0.001
ECG	137,769 (53)	79,244 (47)	51,361 (61)	5164 (69)	< 0.001
Radiological examination	182,540 (71)	108,228 (65)	67,841 (81)	6471 (87)	< 0.001
Treatment information, *n* (%)					
Oxygen administered	56,337 (22)	38,726 (23)	15,689 (19)	1922 (26)	< 0.001
Fluids administered					< 0.001
None	195,911 (76)	136,131 (81)	56,035 (67)	3745 (50)	
≤ 500 ml	35,871 (14)	18,892 (11)	15,182 (18)	1797 (24)	
> 500 ml	26,503 (10)	12,175 (7)	12,407 (15)	1921 (26)	
Medication administered	118,258 (46)	63,987 (38)	48,162 (58)	6109 (82)	< 0.001

Data are presented as mean (SD) if normally distributed or as median (IQR) if skewed. Categorical data are presented as number (%)

*BP* blood pressure; *ED* emergency department; *ECG* electrocardiogram; *GCS* Glasgow Coma Scale; *GP* general practitioner; *IQR* interquartile range; *NRS* numeric rating scale; *SD* standard deviation

Patient outcomes of hospitalized patients triaged non-urgently or urgently can be found in Table 3 (younger patients) and Table 4 (older patients).

**Table 3:** Patient outcomes and discharge status of patients admitted from the ED aged between 18 and 69 years stratified by disease severity as indicated by triage category

	Total cohort (*n = *134,236)	Non-urgent triage categories (*n = *17,058)	(Very) urgent triage categories (*n = *117,178)	*P*
ED LOS in hours, median (IQR)	3.3 (2.0)	3.2 (2.0)	3.3 (2,1)	< 0.001
Discharge status and destination, *n* (%)				< 0.001
Admission to regular ward	120,278 (90)	16,623 (97)	103,655 (89)	
Admission to MCU or CCU	6952 (5)	319 (2)	6633 (6)	
Admission to ICU	7006 (5)	116 (1)	6890 (6)	
Hospital LOS in days, median (IQR)	3.0 (5.0)	2.0 (4.0)	3.0 (5.0)	< 0.001
Mortality, *n* (%)				< 0.001
Discharged alive	130,083 (97)	16,644 (98)	113,429 (97)	
Died in ED or DOA	17 (0)	0 (0)	17 (0)	
Died in hospital before discharge	3536 (3)	158 (1)	3378 (3)	
Not registered	600 (0)	256 (2)	354 (0)	
Return complaints within 7 days, *n* (%)				< 0.001
No return	128,055 (95)	16,164 (95)	111,891 (96)	
Complaints possibly or not related to complaint at reference ED visit	3644 (3)	671 (4)	2973 (3)	
Complaints clearly related to complaint at reference ED visit	2309 (2)	201 (1)	2108 (2)	
Other (e.g., scheduled check-up)	177 (0)	7 (0)	170 (0)	
Not registered	51 (0)	15 (0)	36 (0)	

Data are presented as mean (SD) if normally distributed or as median (IQR) if skewed. Categorical data are presented as number (%)

*CCU* coronary care unit; *DOA* dead on arrival; *ED* emergency department; *ICU* intensive care unit; *IQR* interquartile range; *LOS* length of stay; *MCU* medium care unit, *SD* standard deviation

**Table 4:** Patient outcomes and discharge status of patients admitted from the ED aged 70 years and older stratified by disease severity as indicated by triage category

	Total cohort (*n = *120,034)	Non-urgent triage categories (*n = *15,347)	(Very) urgent triage categories (*n = *104,687)	P
ED LOS in hours, median (IQR)	3.4 (2.1)	3.5 (2.0)	3.4 (2.1)	< 0.001
Discharge status and destination, *n* (%)				< 0.001
Admission to regular ward	110,577 (92)	15,030 (98)	95,547 (91)	
Admission to MCU or CCU	6127 (5)	265 (2)	5862 (6)	
Admission to ICU	3330 (3)	52 (0)	3278 (3)	
Hospital LOS in days, median (IQR)	4.0 (6.0)	4.0 (7.0)	4.0 (6.0)	< 0.001
Mortality, *n* (%)				< 0.001
Discharged alive	110,493 (92)	14,513 (95)	95,980 (92)	
Died in ED or DOA	30 (0)	0 (0)	30 (0)	
Died in hospital before discharge	8975 (7.5)	625 (4)	8350 (8)	
Not registered	536 (0)	209 (1)	327 (0)	
Return complaints within 7 days, *n* (%)				
No return	116,075 (97)	14,796 (96)	101,279 (97)	
Complaints possibly or not related to complaint at reference ED visit	2563 (2)	428 (3)	2135 (2)	
Complaints clearly related to complaint at reference ED visit	1283 (1)	115 (1)	1168 (1)	
Other (e.g., scheduled check-up)	105 (0)	6 (0)	99 (0)	
Not registered	8 (0)	2 (0)	6 (0)	

Data are presented as mean (SD) if normally distributed or as median (IQR) if skewed. Categorical data are presented as number (%)

*CCU* coronary care unit; *DOA* dead on arrival; *ED* emergency department; *ICU* intensive care unit; *IQR* interquartile range; *LOS* length of stay; *MCU* medium care unit, *SD* standard deviation

The median ED LOS of the hospitalized patients was 3.30 (3.30–3.31) h. ED LOS of older hospitalized patients was 3.40 h (3.39–3.41), significantly longer than the 3.30 h (3.29–3.31) of younger patients who were hospitalized. Median hospital LOS was 4.00 days (3.98–4.02) in older patients, compared to 3.00 days (2.98–3.02) in younger patients (P < 0.001).

In-hospital mortality was 2.1% (1.0–3.2%) in the entire cohort of ED patients. Of the admitted patients, in-hospital mortality was three times higher in older patients (7.5% (7.0–8.0%, Table 4)) compared to younger patients [2.6% (1.8–3.5%, Table 3)].

ED patients triaged as urgent were more frequently admitted to specialized units (MCU/CCU/ICU) and had a higher in-hospital mortality compared to patients who were triaged as non-urgent. The ED LOS and hospital LOS were similar within the two subgroups.

### *Association of prolonged ED LOS with hospital LOS* ≥ *3 days*

**Fig. 2:**
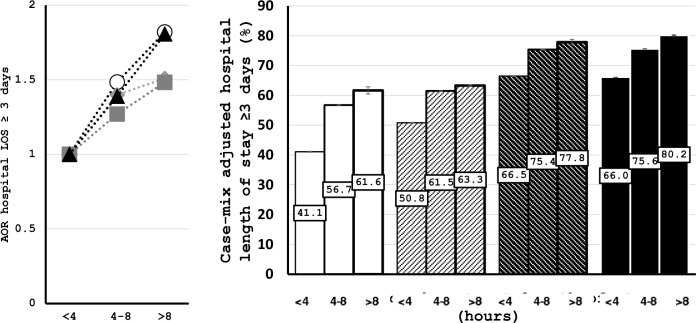
The association between emergency department length of stay and hospital length of stay longer than 3 days. The adjusted odds ratios (left panel) and case-mix-adjusted absolute risk (right panel) for hospital LOS ≥ 3 days as a function of ED LOS are shown, stratified by age < 70 and ≥ 70 years, and non-urgently (blue and green) and urgently (yellow, orange and red)) triaged patients. Error bars represent 95% confidence intervals. Open circles and white bars represent young patients with non-urgent triage categories, diamonds and white bars with black diagonal stripes represent young patients with urgent triage categories, squares and black bars with white diagonal stripes represent old patients with non-urgent triage categories, and triangles and black bars represent old patients with (very) urgent triage categories. *ED* emergency department; *GCS* glasgow coma scale; *LOS* length of stay

In Fig. 2, it is shown that AORs and case-mix adjusted hospital LOS ≥ 3 days increase almost linearly with increasing ED LOS. The impact of prolonged ED LOS was most notable in younger patients who were triaged non-urgently, with an AOR of 1.48 (1.38–1.60) for ED LOS between 4 and 8 h and an AOR of 1.82 (1.40–2.37) for ED LOS > 8 h, compared to patients with an ED LOS < 4 h.

The unadjusted OR’s for hospital LOS ≥ 3 days for the overall cohort and subgroups can be found in Supplemental File 3.

In supplemental file 4, the association between ED LOS as continuous variable and hospital LOS > 3 days is shown, with age as continuous and triage category as ordinal variables. The results in the graphs correspond with the results of the analyses mentioned above.

In Supplemental File 5, it is shown that in the sensitivity analysis without ICU patients’ results are similar. Supplemental file 6 showed that the association between ED and hospital LOS still existed after 16 h.

## Discussion

The main conclusion of the present study is that prolonged ED LOS is associated with an increased hospital LOS, especially in younger patients and  patient who are triaged as non-urgent.

To the best of our knowledge, this study is the first to show that ED-LOS is associated with prolonged hospital LOS. Previous studies, in healthcare systems with significantly longer ED LOS [1–6], found an association between ED-LOS and mortality, except for several studies [23, 24], including a recent French study [25]. Although mortality is a different outcome, our study further corroborates the detrimental effects of prolonged ED-LOS. One study showed that an increasing number of patients is dying in the ED instead of in a familiar setting, which was associated with an increased ED LOS [26]. Given the observations in the present study, this increased ED LOS could then also increase hospital LOS for the other ED patients.

The relationship between ED LOS and hospital LOS remained significant even in the extended time frames (Supplemental File 6). In addition, 6 of the 9 Bradford-Hill criteria [27] fully applied to our study, and 3 partially did, so it is possible that this is a causal relation (Supplemental File 7).

Although the reason for the positive association between ED LOS and hospital LOS longer than 3 days remains to be elucidated, it is possible that delayed recognition of disease severity results in delayed initiation of treatment. More importantly, it is known that delayed transfer to an appropriate level of care is associated with adverse outcomes [17, 28], suggesting that ED LOS also affects hospital costs. However, even after excluding ICU patients, a significant association between ED LOS and prolonged hospital LOS persisted, suggesting that it is not only the critically ill in which prolonged ED-LOS is associated with prolonged hospital LOS but also in less ill patients admitted to a normal ward.

If the found association would be a causal relation and we multiply the average longer hospital admission days in ED patients with an ED LOS > 4 h, with the costs per hospital admission day, €173 million associated with prolonged hospital LOS could be prevented if interventions could reduce prolonged ED LOS (Supplemental File 8).

In contrast to our initial hypothesis, we found that patients who were triaged non-urgently had the highest odds for prolonged hospital LOS. Possible explanations could be that non-urgent patients who are admitted were undertriaged [29], as many of these patients who died had high risk and nonspecific presenting complaints, possibly complicating recognition of disease severity and consequently delayed initiation of treatment. Especially during times of overcrowding or understaffing this could have had detrimental effects [30] although we did not measure this in the present study. Another explanation could be that older patients who are triaged urgently have already a high baseline hospital LOS (Fig. 2) and that other factors than prolonged ED LOS are more important. Finally, in older patients, the increased LOS may have been caused by a night on a hard cot with insufficient monitoring and care and sleep disturbance in the ED as this has been shown to have adverse effects, even in patients with low disease severity [31, 32]. This unexpected finding emphasizes the importance of investigation of the association between ED LOS in subgroups rather than in the total cohort.

Although our study has its strengths such as the large representative sample and analyses per subgroup, there are several limitations. First, the NEED lacks data on patients’ comorbidities. To overcome this, we utilized variables linked to comorbidities and complexity, as done previously [18, 19, 22]. Data on ED crowding at the time of patient arrival were not available so we could not assess whether ED LOS indeed increased when crowding occurred. Secondly, the observational nature of the study introduces the possibility of information bias. Despite implementing automated data collection processes and rigorous validation measures, the NEED remains susceptible to human errors in documentation. However, data transfers are automated and subject to meticulous validation, ensuring the inclusion of only reliably registered variables from the hospital information system [15].

## Conclusion

In the Dutch healthcare system, with relatively short ED LOS, prolonged ED LOS is associated with prolonged hospital LOS, especially in younger patients who are triaged non-urgently. Future research should clarify whether this is a causal relationship and whether the association is different in the lowest triage category (blue) and second lowest triage category (green). In addition, it should be investigated whether reduction of ED LOS improves outcomes of ED patients.

## Supplementary Information

Below is the link to the electronic supplementary material.

Supplementary file1 (DOCX 181 KB)

## Data Availability

The data that support the findings of this study are available from the NEED foundation (www.stichting-need.nl) but restrictions apply to the availability of these data, which were used under license for the current study, and so are not publicly available. Data are, however, available from the authors upon reasonable request and with permission of the NEED foundation.

## Abbreviations

CCU: Coronary care unit
ED: Emergency department
ICU: Intensive care unit
IQR: Interquartile range
MCU: Medium care unit
NEED: Netherlands Emergency department Evaluation Database
LOS: Length of stay
OR: Odds ratio
SD: Standard deviation

## References

[1] Lauque D, Khalemsky A, Boudi Z, Östlundh L, Xu C, Alsabri M et al (2022) Length-of-stay in the emergency department and in-hospital mortality: a systematic review and meta-analysis. J Clin Med 12:32 36614835 10.3390/jcm12010032 PMC9821325

[2] Lee KS, Min HS, Moon JY, Lim D, Kim Y, Ko E et al (2022) Patient and hospital characteristics predict prolonged emergency department length of stay and in-hospital mortality: a nationwide analysis in Korea. BMC Emerg Med 22:183 36411433 10.1186/s12873-022-00745-y PMC9677700

[3] Verma A, Shishodia S, Jaiswal S, Sheikh WR, Haldar M, Vishen A et al (2021) Increased length of stay of critically ill patients in the emergency department associated with higher in-hospital mortality. Indian J Crit Care Med 25:1221–1225 34866817 10.5005/jp-journals-10071-24018 PMC8608642

[4] Paton A, Mitra B, Considine J (2019) Longer time to transfer from the emergency department after bed request is associated with worse outcomes. Emerg Med Australas 31:211–215 30129706 10.1111/1742-6723.13120

[5] Mowery NT, Dougherty SD, Hildreth AN, Holmes JHT, Chang MC, Martin RS et al (2011) Emergency department length of stay is an independent predictor of hospital mortality in trauma activation patients. J Trauma 70:1317–1325 21817968 10.1097/TA.0b013e3182175199

[6] Jones S, Moulton C, Swift S, Molyneux P, Black S, Mason N et al (2022) Association between delays to patient admission from the emergency department and all-cause 30-day mortality. Emerg Med J 39:168–173 35042695 10.1136/emermed-2021-211572

[7] Emergency Medicine Practice Committee (2016) ACEP. Emergency department overcrowding: high impact solutions. ACEP, Irving

[8] Bernstein SL, Aronsky D, Duseja R, Epstein S, Handel D, Hwang U et al (2009) The effect of emergency department crowding on clinically oriented outcomes. Acad Emerg Med 16:1–10 19007346 10.1111/j.1553-2712.2008.00295.x

[9] Sun BC, Hsia RY, Weiss RE, Zingmond D, Liang LJ, Han W et al (2013) Effect of emergency department crowding on outcomes of admitted patients. Ann Emerg Med 61:605–611 23218508 10.1016/j.annemergmed.2012.10.026 PMC3690784

[10] Wang H, Kline JA, Jackson BE, Robinson RD, Sullivan M, Holmes M et al (2017) The role of patient perception of crowding in the determination of real-time patient satisfaction at Emergency Department. Int J Qual Health Care 29:722–727 28992161 10.1093/intqhc/mzx097

[11] Tsai CL, Rowe BH, Cydulka RK, Camargo CA Jr (2009) ED visit volume and quality of care in acute exacerbations of chronic obstructive pulmonary disease. Am J Emerg Med 27:1040–1049 19931748 10.1016/j.ajem.2008.07.034

[12] Landelijk Netwerk Acute Zorg (2023) SEH-stops in kwartaal 2 van 2023 [ED stops in quarter 2 of 2023]. [Internet]. Available from: https://www.lnaz.nl/nieuws/seh-stops-in-kwartaal-2-van-2023

[13] van der Linden C, Reijnen R, Derlet RW, Lindeboom R, van der Linden N, Lucas C et al (2013) Emergency department crowding in The Netherlands: managers’ experiences. Int J Emerg Med 6:41 24156298 10.1186/1865-1380-6-41 PMC4016265

[14] Foley M, Kifaieh N, Mallon WK (2011) Financial impact of emergency department crowding. West J Emerg Med 12:192–197 21691525 PMC3099606

[15] Netherlands Emergency department Evaluation Database (2025) Dé discipline overstijgende kwaliteitsregistratie van Spoedeisende hulp afdelingen [The quality registry of emergency departments]. [Internet]. Available from: www.stichting-need.nl

[16] Aarts F (2022) SEH-artsen: 'Bijna 1000 patiënten overlijden onnodig door lange wachttijd' [ER doctors: 'Nearly 1000 patients die unnecessarily due to long wait']. https://nl.linkedin.com/posts/frits-aarts-17207814_seh-artsen-bijna-1000-pati%C3%ABnten-overlijden-activity-6945293433993355264-eUsM?trk=public_profile_like_view. Accessed 22 June 2022

[17] Chalfin DB, Trzeciak S, Likourezos A, Baumann BM, Dellinger RP (2007) Impact of delayed transfer of critically ill patients from the emergency department to the intensive care unit. Crit Care Med 35:1477–1483 17440421 10.1097/01.CCM.0000266585.74905.5A

[18] van der Veen D, Remeijer C, Fogteloo AJ, Heringhaus C, de Groot B (2018) Independent determinants of prolonged emergency department length of stay in a tertiary care centre: a prospective cohort study. Scand J Trauma Resusc Emerg Med 26:81 30236125 10.1186/s13049-018-0547-5 PMC6148782

[19] van der Veen D, Heringhaus C, de Groot B (2016) Appropriateness, reasons and independent predictors of consultations in the Emergency Department (ED) of a Dutch tertiary care center: a prospective cohort study. PLoS ONE 11:e0149079 26894273 10.1371/journal.pone.0149079 PMC4760948

[20] Shmueli G (2010) To explain or to predict? Stat Sci 25(289–310):22

[21] Vittinghoff E, McCulloch CE (2007) Relaxing the rule of ten events per variable in logistic and Cox regression. Am J Epidemiol 165:710–718 17182981 10.1093/aje/kwk052

[22] Raven W, van den Hoven EMP, Gaakeer MI, Ter Avest E, Sir O, Lameijer H et al (2022) The association between presenting complaints and clinical outcomes in emergency department patients of different age categories. Eur J Emerg Med 29:33–41 34406137 10.1097/MEJ.0000000000000860

[23] Chong CP, Haywood C, Barker A, Lim WK (2013) Is Emergency Department length of stay associated with inpatient mortality? Australas J Ageing 32:122–124 23773253 10.1111/j.1741-6612.2012.00651.x

[24] Ashkenazi I, Gefen L, Hochman O, Tannous E (2021) The 4-hour target in the emergency department, in-hospital mortality, and length of hospitalization: a single center-retrospective study. Am J Emerg Med 47:95–100 33794476 10.1016/j.ajem.2021.03.049

[25] Balen F et al (2023) Impact of emergency department length of stay on in-hospital mortality: a retrospective cohort study. Eur J Emerg Med. 10.1097/MEJ.0000000000001079 37788143 10.1097/MEJ.0000000000001079

[26] Gianfranco Cervellin G, Casagranda I, Ricci G, Mezzocolli I, Paolillo C, Rossi R, Bellone A, Guzzetti S, Giostra F, Rastelli G, Cavazza M (2017) Unavoidable deaths in the Italian Emergency Departments. Results of a ten-year survey. A mirror of substantial social changes, or a warning for a hospital-system pathology? Emerg Care J 13:6718

[27] Hill AB (1965) The environment and disease: association or causation? Proc R Soc Med 58:295–300 14283879 10.1177/003591576505800503 PMC1898525

[28] De Groot B, de Deckere ERJT, Flameling R, Sandel MH, Vis A (2012) Performance of illness severity scores to guide disposition of emergency department patients with severe sepsis or septic shock. Eur J Emerg Med 19(5):316–322 22008587 10.1097/MEJ.0b013e32834d6efb

[29] Grande-Ratti MF, Esteban JA, Mongelos D, Díaz MH, Giunta DH, Martinez BJ (2020) Undertriage as quality of care parameter in an emergency department. Rev Med Chil 148:602–610 33399753 10.4067/S0034-98872020000500602

[30] Tarnow-Mordi WO, Hau C, Warden A, Shearer AJ (2000) Hospital mortality in relation to staff workload: a 4-year study in an adult intensive-care unit. Lancet 356:185–189 10963195 10.1016/s0140-6736(00)02478-8

[31] Roussel M, Teissandier D, Yordanov Y, Balen F, Noizet M, Tazarourte K, Bloom B, Catoire P, Berard L, Cachanado M, Simon T, Laribi S, Freund Y, for the FHU IMPEC−IRU SFMU Collaborators (2023) Overnight stay in the Emergency Department and mortality in older patients. JAMA Intern Med 183(12):1378–1385 37930696 10.1001/jamainternmed.2023.5961 PMC10628833

[32] Miró O, Aguiló S, Alquézar-Arbé A, Fernández C, Burillo G, GuzmánMartínez S, MartínezLarrull ME, Periago AB, Molinas CLA, Rangel Falcón C, BaladoDacosta P, Chávez Flores RC, Navarro Calzada J, FrageroBlesa EM, Palomero Martín MA, CobosRequena A, Fuentes L, Lobo Cortizo I, González Garcinuño P, BóvedaGarcía M, Rivas Del Valle P, Benavent Campos R, Castro Jiménez V, Abad Cuñado V, Trejo Gutiérrez O, del Mar Sousa Reviriego M, Roussel M, González del Castillo J (2024) Overnight stay in Spanish emergency departments and mortality in older patients. Internal Emerg Med. 10.1007/s11739-024-03660-1

