# Supplementary material for: The association between emergency department length of stay and hospital length of stay: an observational multi-centre cohort study
Source: Intern Emerg Med. 2025 May 26;21(2):631–43. doi: 10.1007/s11739-025-03964-w (PMC13061830; doi:10.1007/s11739-025-03964-w)
Supplement: Supplementary file 1 — Supplementary file1 (DOCX 181 KB) [file 11739_2025_3964_MOESM1_ESM.docx]

**SUPPLEMENTAL FILE 1: Data definitions in the NEED**

**GENERAL DATA OF PARTICIPATING EMERGENCY DEPARTMENTS**
In the NEED, several hospitals and the emergency department characteristics are registered once a year.

**1. Which hospital.** Coding: <Hospital Code>.
**2. Type of hospital**. Urban = 0. Urban (STZ = Foundation of top clinical hospitals) teaching hospital = 1. Academic Medical Centre = 2.
**3. Number of patients in the region of the relevant hospital**. This information can be obtained for each participating hospital by the NZa (was once used to calculate the hospital budgets).
**4. Emergency physicians.** a. Number of emergency physicians working at the emergency room in the hospital on the first of January of the year in question. b. Emergency physicians in fulltime equivalent. c. Number of residents in emergency medicine on the first of January of the year in question. d. Number of residents not in training on the first of January of that year.
**5. Presence of emergency physicians.** a. Non-24/7 presence by emergency physicians = 0. Minimum of 1 emergency physician 24 hours a day, 7 days a week = 1. b. How many emergency physicians, residents in emergency medicine and residents not in training are working average at the emergency department daily for patient care. Number per shift. The number of shifts for emergency physicians also must be defined.
**6. Number of trauma surgeons employed by the hospital (so NO general surgeons).** Trauma surgeons in FTE.
**7. Number of internists in acute medicine employed by the hospital. (So NOT general internists).**
**8a. Trauma center** No = 0. Level 2 = 1. Level 1 = 2.
**8b. Intensive care in hospital.** Not present = 0. Present = 1.
**9. Medium Care (or high care) present in hospital.** Not present = 0. Present = 1.
**10. Number of visits per year on the emergency department (so NOT the number of emergency patients per year).** This only accounts for the number of patients at the emergency department. If a separate emergency cardiac care is present or GP clinic is integrated, these patient visits are counted separately.
**11. Number of emergency department visits per year.** N.
**12. General Practitioner clinic integrated within the emergency department.** No = 0. Yes = 1.
**13. Emergency cardiac care present AT or NEXT to the emergency department.** Not present = 0. Present = 1
**14. Quality indicators by the Dutch Society of Emergency Physicians (DSEP (NVSHA).** See also the NVSHA website. [www.nvsha.nl](http://www.nvsha.nl).

**Quality indicators for Acute Coronary Syndrome (ACS).
1. Are patients with (suspected) ACS treated at your emergency department?** Indicate whether any patients are suspected for acute coronary syndrome at your emergency department. Answer: No = 0. Yes = 1.  **2. For patients with (suspected) ACS, do you have a protocol about administration of aspirin?** Indicate whether a protocol is used at the emergency department for the administration of aspirin in cases of suspected acute coronary syndrome. Mention which protocol. Answer: No = 0. Yes = 1.  **3. Time for administration of aspirin to patients with (suspected) ACS registered at the emergency room?** Specify whether the exact time of administration of aspirin given to patients with suspicion of acute coronary syndrome is registered at the emergency department. This can be a written or electronic registration. Answer: No = 0. Yes = 1.

**Quality indicator Complication registration**.
*A complication is an unintended and undesired event or condition during or following medical treatment which is harmful to the health of the patient needing change in medical treatment, or if irreversible health damage exists.*
**1. Complication registrations**
Is a complication registration used at the emergency department? Only one answer is possible. If this question is answered with "no", the other questions with indicator "Triage on Emergency Department" do not apply. You can continue to answer the questions with the next indicator. Answer: No = 0. Yes = 1.
**2. Complication meeting**
A complication meeting at least four times a year at the emergency room? Answer only yes or no. Answer: No = 0 Yes = 1.
**3. Complication Registration**
Here we would like you to briefly specify which CR you are using. If possible, you can attach a copy of the list of complications registered. Please note: This does not concern completed lists with numbers of complications. Free text: …

**Quality indicator child abuse / domestic violence.**
**1.Presence of child abuse protocols**
A written child abuse protocol used at the emergency department which meets the following minimum competencies? A. A multidisciplinary child abuse team is active in the hospital. B. Employees at the emergency department are trained in identifying child abuse. Answer: Yes, to both: 3 A yes, B no: 2 A no, B yes: 1 No = 0.
**2. Screening instrument**
Does the ED use a screening instrument to signal child abuse, such as the SPUTOVAMO form or a derivative? Answer: No = 0. Yes = 1. Which screening instrument is used at the emergency room? (multiple answers possible): SPUTOVAMO form. No = 0. Yes = 1 Top - toe examination. No = 0. Yes = 1. Otherwise, describe:
**3. Number of completed screening documents.**
What is the number of completed screening documents at the emergency department, in relation to the total number of children up to and including 18 years old at the emergency department?
**n** Number of completed screening documents at the ED in January 2011
**N** Total number of children up to and including 18 years at the emergency room in January 2011
**n / N * 100%** Screening document percentage
**4. Protocol "parent notifications"**
Is a protocol used at the emergency department for the reporting of children as victims of domestic violence / suicidal attempt / auto mutilation / excessive alcohol and / or drug use? Answer: No = 0. Yes = 1. Notifications:

**Quality indicator pain relief.
1. Pain scoring systems** Is a pain scoring system used at the emergency department? Answer: No = 0. Yes = 1. If yes, which scoring system: <Free text> **2. Time registration of pain relief.** Is the exact time recorded when pain relief is given in the emergency room? Answer: No = 0. Yes = 1.  **3. Pain protocols** Is a pain protocol used at the emergency room? Answer: No = 0. Yes = 1. Comments <Free text>
**Quality indicator Sepsis**.  **1.Sepsis protocol** Is a sepsis protocol used at the emergency department? Answer: No=0. Yes=1. If yes, which protocol <Free text> **2. Screening document Is a screening document used for sepsis in the emergency room?** Answer: No=0. Yes=1. If yes, which document: <Free text>
**3. Time registration for administering of antibiotics to patients with sepsis** Is time of administering of antibiotics to patients with sepsis registered at the emergency department? Answer No=0. Yes=1. Comments <Free text>

**Quality indicator Procedural sedation and analgesia (PSA)
PATIENT RECORDS.**
The following basic principles are used: 1) Facts are recorded, e.g. times. Derived variables are generated afterwards, such as duration of stay. 2) Continuous variables are not categorized because this will lead to data loss. 3) Only create categories for coding, considering a remaining category "other" and the possibility to indicate "unknown" The following data is collected from all patients:  **Demographic information.** In general, the social service number (BSN) and date of birth are registered for each patient. In addition, a unique code is registered for each emergency room visit.  **1.Age** Age in years at the time of registration at the emergency registration desk.  **2. Sex.** Male or female. Code: Female = 0. Male = 1  **3. Date and time Registration of ED presentation.** DD-MM-YEAR-HOUR-MIN. Transport to the hospital.  **4. Type of arrival at the hospital.** Arrival at the emergency room by ambulance or by own transport (i.e. walking, bicycle / moped or public transport). Encoding: Own transport = 0. Arrival with ambulance = 1. 9 = Unknown  **5. Referral status**. Self-referrer or referred by GP or another specialist. Encoding: Self-referrer = 0. Referred by physician = 1. Referred by specialist = 2. 9 = Unknown. For variable 4 and 5, more variables are possible. This still has to be discussed and agreed with the participating hospitals. Example: Anyone who enters by ambulance after a 112-call, would that be a self-referrer?

**General information in the ED.
6. Triage category**. Triage category according to Manchester triage system upon arrival of patient. Coding: Blue = 1, green = 2, yellow = 3, orange = 4, red = 5. If the Boston triage system is used, the corresponding categories 1 to 5 will be used. If a Dutch triage system is used, it will be entered. **7. Main specialism or supervisor**. In case this is not known, the specialty will be recorded which admits the patient. Coding: emergency physician = 0. Surgery = 1. Internal medicine (or super specialism of internal medicine for example gastroenterologist or vascular medicine) = 2. Cardiology = 3. Neurology = 4. Urology, ENT or ophthalmology = 5. Pediatrics = 6. Other = 7.  **8. Presenting complaint according to MTS, NTS, etc triage system.** So not the entry complaint according to the registration desk employee. Coding: Complaint 1 = 1, complaint 2 = 2, ........... Complaint 52 = complaint 52.  **9. Resuscitation in shock (trauma) room.** Coding: Treatment in standard treatment room = 0. Shock room / trauma room = 1. The definition of a shock room still needs to be determined by the participating emergency departments. They need to agree whether, for example, thrombolysis is part of this or not. The exact conditions of this room must be specified?  **10. Initial vital signs at the time of triage or in the ED treatment room.** So NOT mentioned on arrival at the treatment room. This variable only needs to be registered if it has been measured. If not measured, the space is left empty. Oxygen saturation as measured with a pulse oximeter used in the hospital (percentages without oxygen). If measured when oxygen is given, note the number of liters / min of oxygen. Systolic and diastolic blood pressure (mmHg).Heart rate measured with pulse oximeter (beats / min.).Temperature measured with an ear thermometer (degrees Celsius). Glascow coma scale (EMV score). A separate variable is made of each vital parameter, with limit values and units. The Early Warning Score can then be calculated.  **11a. Additional blood tests on the emergency room.** If blood has not been obtained and sent for analyses, a 0 will be entered. If blood has been sent for analyses, a 1 will be entered.  **11b. If blood has been obtained, the following values are entered.** Carefully check whether the units at all EDs are the same! Blood gas obtained. 0 = No. 1 = Yes. If yes: Venous = 0. Arterial = 1. pH …PO2 (KPa), PCO2 (KPa), Bicarbonate (mmol / L), B.E. ... Biochemistry: Sodium (mmol / L), K (mmol / L), Creatinine (μmol / L) Urea (mmol / L), ASAT (U / L), ALAT (U / L), yGT (U / L), AF (U / L), LDH (U / L), CK (U / L), High sensitive Troponin (ng / L), CRP (mg / L), Pro-BNP (mg / L), Procalcitonin (mg / L), Lactate (mmol / L) Hematology: Hb (mmol / L), Leucocytes ( x109 / L), Platelets ( x1012 / L), D-dimer (mg / L), INR Toxicology: Ethanol (mg / L), Paracetamol (mg / L) *Blood cultures collected at the emergency room and sent for cultures*: 0 = No. 1 = Yes. Blood cultures should be at least one set (i.e. an aerobic and an anaerobic*). Urine collected at the emergency room and send for cultures*: 0 = No. 1 = Yes. *Urinary sediment collected at the emergency room:* 0 = No. 1 = Yes. *Urine toxicological analysis performed at the emergency department*: 0 = No. 1 = Yes.  **12a. Additional X-ray diagnostics at the emergency room**.
If no X-ray diagnostics has been performed at the emergency room, a 0 is entered. If a type of X-ray diagnostics has been performed at the emergency room, a 1 is entered. Definition: 0 should also be entered, if the patient is sent for an X-ray by the GP, and afterwards sent to the Emergency Department.  **12b. If X-ray diagnostics have been performed, the type of examination must be recorded**. Conventional X-ray, extremity. 0 = No. 1 = Yes. Conventional X-ray, divers (including chest X-ray). 0 = No, 1 = Yes. Abdominal ultrasound (including FAST). 0 = No, 1 = Yes. Ultrasound for deep vein thrombosis (DVT). 0 = No, 1 = Yes. Ultrasound, divers. 0 = No, 1 = Yes. Head CT. 0 = No, 1 = Yes. CT-Cervical spine. 0 = No, 1 = Yes. CT pulmonary embolism. 0 = No, 1 = Yes. CT aorta. 0 = No, 1 = Yes. CT chest-abdomen (trauma). 0 = No, 1 = Yes. Patients can receive multiple examinations at the emergency room.  **13. Number of consultations at the emergency room.** Coding: No consultations = 0. A consultation = 1. Two consultations = 2. Three consultations = 3, etc.

**Outcomes
14. Emergency department lengths of stay.** Duration in minutes. Discharge time of the emergency room minus the registration time at the emergency room. (So NOT the announcement or triage time).  **15. Discharge destination (disposition)** Home, to general ward, medium care (MC) or Coronary Care Unit (CCU) or intensive care (ICU). Died in ED. Transfer to another hospital. Scheduled outpatient clinic after ED visit and related to ED visit.  **16. Hospital length of stay.** In days. Discharge date minus recording date. If a patient at the emergency room is discharged home, the hospital duration is 0 days. If people are registered before midnight and leave the ED after midnight, this should be accounted as 0 days!  **17. Hospital mortality.** Coding: Leaving the hospital alive = 0. Died in ED or brought in dead (for example during CPR) = 1. Died in hospital before discharge = 2.  **18. Return to ED with medical problem / complaints.** Coding: No return with medical problems within 7 days after registration time on ED = 0. Return to the ED within a week after discharge with a medical problem which may or may not be related to the previous ED visit = 1. Return to the ED within a week after discharge with a medical problem which is clearly related to the previous ED visit = 2. Otherwise = 3  **19. ICD-10 code and diagnosis after discharge from the emergency room and hospital.
20. Diagnosis Treatment Code (DBC or DOT).**

**SUPPLEMENTAL FILE 2: Patient characteristics.**

| **Table S1** Characteristics of patients in the emergency department stratified by age | | | |
| --- | --- | --- | --- |
|  | Total cohort  (n = 718,358) | Age <70  (n = 501,147) | Age ≥70  (n = 217,211) |
| **Demographics** |  |  |  |
| Age, mean (SD) | 51 (25) | 39 (21) | 79 (6) |
| Male sex, n (%) | 373,726 (52) | 263,755 (53) | 109,971 (51) |
| Previous ED attendances, median (IQR) | 2 (2) | 1 (2) | 2 (3) |
| **Patient pathway, n (%)** |  |  |  |
| Type of hospital |  |  |  |
| Academic | 165,442 (23) | 127,551 (26) | 37,891 (17) |
| Urban | 552,916 (77) | 373,596 (75) | 179,320 (83) |
| Type of arrival |  |  |  |
| Private transport | 448,804 (63) | 352,166 (70) | 96,638 (44) |
| Ambulance | 233,736 (33) | 127,011 (25) | 106,725 (49) |
| Not registered | 35,818 (5) | 21,970 (4) | 13,848 (6) |
| Referral source |  |  |  |
| Self-referral | 239,053 (33) | 171,754 (34) | 67,299 (31) |
| GP | 375,076 (52) | 251,546 (50) | 123,530 (57) |
| Specialist | 84,447 (12) | 63,676 (13) | 20,771 (10) |
| Not registered | 19,782 (3) | 14,171 (3) | 5,611 (3) |
| **Top 10 presenting complaints, n (%)** |  |  |  |
| Extremity complaints | 137,552 (19) | 109,798 (22) | 27,754 (13) |
| Feeling unwell | 108,656 (15) | 61,189 (12) | 47,467 (22) |
| Abdominal pain | 69,096 (10) | 54,382 (11) | 14,714 (7) |
| Shortness of breath | 59,586 (8) | 32,578 (7) | 27,008 (12) |
| Chest pain | 52,191 (7) | 34,117 (7) | 18,074 (8) |
| Trauma, severe | 32,915 (5) | 24,309 (5) | 8,606 (4) |
| Wounds | 26,156 (4) | 21,669 (4) | 4,487 (2) |
| Fallen | 15,577 (2) | 8,842 (2) | 6,735 (3) |
| Collapse | 13,263 (2) | 6,760 (1) | 6,503 (3) |
| Vomiting, diarrhea | 7,598 (1) | 5,139 (1) | 2,459 (1) |
| **Triage category, n (%)** |  |  |  |
| Green/blue | 184,214 (26) | 141,814 (28) | 42,400 (20) |
| Yellow | 364,899 (51) | 246,441 (49) | 118,458 (55) |
| Orange | 131,751 (18) | 85,806 (17) | 45,945 (21) |
| Red | 15,661 (2) | 10,050 (2) | 5,611 (3) |
| Not registered | 21,833 (3) | 17,036 (3) | 4,797 (2) |
| **Attending physician, n (%)** |  |  |  |
| ED physician | 86,325 (12) | 66,193 (13) | 20,132 (9) |
| Surgical specialty | 210,419 (30) | 162,484 (32) | 47,935 (22) |
| Medical specialty | 405,412 (56) | 258,160 (52) | 147,252 (68) |
| Not registered | 16,202 (2) | 14,310 (3) | 1,892 (1) |
| **Number of consultations in ED, n (%)** |  |  |  |
| 0 | 402,966 (56) | 284,955 (57) | 118,011 (54) |
| 1 | 212,110 (30) | 140,344 (28) | 71,766 (33) |
| 2 | 83,035 (12) | 60,436 (12) | 22,599 (10) |
| ≥3 | 10,998 (2) | 7,024 (1) | 3,974 (2) |
| Not registered | 9,249 (1) | 8,388 (2) | 861 (0) |
| **Vital signs at ED presentation** |  |  |  |
| Registered vital signs, n (%) |  |  |  |
| None registered | 197,719 (28) | 167,241 (33) | 30,478 (14) |
| A few registered | 198,124 (28) | 141,702 (28) | 56,422 (26) |
| All registered | 322,515 (45) | 192,204 (38) | 130,311 (60) |
| Respiratory rate (per minute), median (IQR) | 16 (6) | 16 (6) | 17 (6) |
| Oxygen saturation (%), median (IQR) | 98 (3) | 98 (4) | 97 (3) |
| Heart rate (per minute), median (IQR) | 84 (28) | 86 (27) | 82 (26) |
| Systolic BP (mmHg), mean (SD) | 140 (28) | 136 (25) | 148 (30) |
| Diastolic BP (mmHg), mean (SD) | 82 (17) | 83 (16) | 80 (18) |
| GCS, n (%) |  |  |  |
| Not registered | 564,654 (79) | 404,707 (81) | 159,947 (74) |
| Registered, < 15 | 12,786 (2) | 7,447 (2) | 5,339 (3) |
| Registered, = 15 | 140,918 (20) | 88,993 (18) | 51,925 (24) |
| NRS Pain score, n (%) |  |  |  |
| Not registered or 0 | 522,654 (73) | 358,704 (72) | 163,950 (76) |
| 1-3 | 90,689 (13) | 61,950 (12) | 28,739 (13) |
| 4-6 | 69,225 (10) | 51,619 (10) | 17,606 (8) |
| 7+ | 35,790 (5) | 28,874 (6) | 6,916 (3) |
| Temperature (°C), mean (SD) | 36.9 (1.0) | 37.0 (1.0) | 36.9 (0.9) |
| **Diagnostic testing, n (%)** |  |  |  |
| Laboratory tests | 444,976 (62) | 271,774 (54) | 173,202 (80) |
| ECG | 231,765 (32) | 124,960 (25) | 106,805 (49) |
| Radiological examination | 413,989 (58) | 265,929 (53) | 148,060 (68) |
| **Treatment information, n (%)** |  |  |  |
| Oxygen administered | 83,046 (12) | 43,521 (9) | 39,525 (18) |
| Fluids administered |  |  |  |
| None | 633,571 (88) | 449,761 (90) | 183,810 (85) |
| ≤500 ml | 52,862 (7) | 32,065 (6) | 20,797 (10) |
| >500 ml | 31,925 (4) | 19,321 (4) | 12,604 (6) |
| Medication administered | 224,741 (31) | 151,729 (30) | 73,012 (34) |
| Data are presented as mean (SD) if normally distributed or as median (IQR) if skewed. Categorical data are presented as number (%).  Abbreviations: BP, blood pressure; ED, emergency department; ECG, electrocardiogram; GCS, Glasgow Coma Scale; GP, general practitioner; IQR, interquartile range; NRS, numeric rating scale; SD, standard deviation. | | | |

**SUPPLEMENTAL FILE 3: Unadjusted Odds Ratio’s for hospital LOS ≥3 days.**

| **Table S2.** Unadjusted Odds Ratio’s for the association between ED LOS and hospital LOS of 3 or more days by different age groups and disease severity as indicated by triage category | | | | |
| --- | --- | --- | --- | --- |
|  | **ED LOS <4 hours^a^** | **ED LOS 4-8 hours** | **ED LOS >8 hours** | **Number** |
| **Total cohort** | 1.00 | 1.60 (1.57-1.63) | 1.90 (1.82-2.02) | 255,566 |
|  |  |  |  |  |
| **Age (years)** |  |  |  |  |
| <70 | 1.00 | 1.57 (1.54-1.61) | 1.73 (1.62-1.86) | 135,123 |
| ≥70 | 1.00 | 1.59 (1.55-1.64) | 2.06 (1.90-2.23) | 120,443 |
|  |  |  |  |  |
| **Triage categories** |  |  |  |  |
| Non-urgent | 1.00 | 1.77 (1.69-1.86) | 2.04 (1.69-2.47) | 31,685 |
| (Very) urgent | 1.00 | 1.58 (1.55-1.61) | 1.88 (1.79-1.99) | 219,667 |
|  |  |  |  |  |
| **Age*triage categories** |  |  |  |  |
| <70*non-urgent | 1.00 | 1.88 (1.75-2.01) | 2.30 (1.79-2.96) | 16,620 |
| <70*(very) urgent | 1.00 | 1.52 (1.51-1.58) | 1.67 (1.55-1.79) | 116,011 |
| ≥70*non-urgent | 1.00 | 1.58 (1.44-1.67) | 1.76 (1.30-2.37) | 15,065 |
| ≥70*(very) urgent | 1.00 | 1.60 (1.55-1.64) | 2.08 (1.91-2.26) | 103,656 |
| ^a^ Reference category in the regression analyses.  Abbreviations: ED, emergency department; LOS, length of stay. | | | | |

**SUPPLEMENTAL FILE 4: The association between Emergency Department (ED) length of stay (LOS; as continuous variable) and hospital length of stay >3 days.**


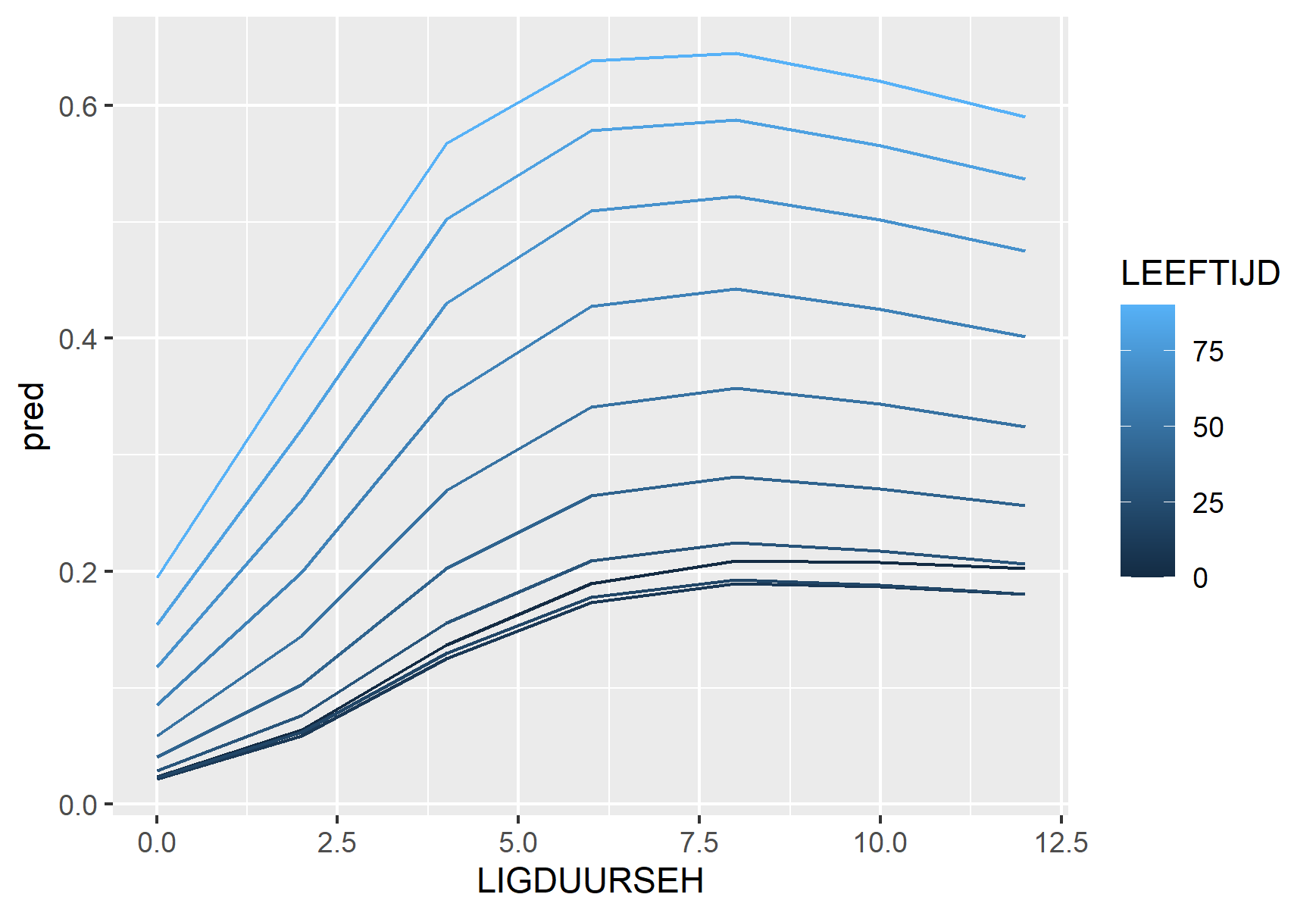


Emergency Department Length of Stay (hrs)

Predicted probability for Hospital

Length of Stay ≥3 days

**SUPPLEMENTAL FILE 5: Sensitivity analysis without ICU patients**

| **Table S3** Adjusted Odds Ratio’s for the association between ED LOS and hospital LOS of 3 or more days in patients admitted through the ED to a general ward, by different age groups and disease severity as indicated by triage category | | | | |
| --- | --- | --- | --- | --- |
|  | **ED LOS <4 hours^a^** | **ED LOS 4-8 hours** | **ED LOS >8 hours** | **Number** |
| **Total cohort** | 1.00 | 1.40 (1.38-1.43) | 1.65 (1.56-1.75) | 228,074 |
|  |  |  |  |  |
| **Age (years)** |  |  |  |  |
| <70 | 1.00 | 1.43 (1.39-1.46) | 1.58 (1.46-1.70) | 118,759 |
| ≥70 | 1.00 | 1.37 (1.33-1.41) | 1.74 (1.60-1.90) | 109,315 |
|  |  |  |  |  |
| **Triage categories** |  |  |  |  |
| Non-urgent | 1.00 | 1.37 (1.30-1.45) | 1.64 (1.34-2.01) | 30,924 |
| (Very) urgent | 1.00 | 1.40 (1.37-1.43) | 1.66 (1.56-1.76) | 197,140 |
|  |  |  |  |  |
| **Age*triage categories** |  |  |  |  |
| <70*non-urgent | 1.00 | 1.47 (1.36-1.58) | 1.80 (1.37-2.35) | 16,185 |
| <70*(very) urgent | 1.00 | 1.41 (1.37-1.45) | 1.55 (1.43-1.68) | 102,574 |
| ≥70*non-urgent | 1.00 | 1.23 (1.16-1.36) | 1.45 (1.06-1.98) | 14,749 |
| ≥70*(very) urgent | 1.00 | 1.38 (1.34-1.43) | 1.78 (1.63-1.94) | 94,566 |
| ^a^ Reference category in the regression analyses. In the final model we adjusted for the following confounders: age, sex, triage category, top 10 presenting complaint, number of consultations in the ED, GCS, laboratory tests, and radiological tests.  Abbreviations: ED, emergency department; GCS, glasgow coma scale; LOS, length of stay. | | | | |

**SUPPLEMENTAL FILE 6: Association ED LOS across five categories and hospital lengths of stay ≥3 days.**

**Crude model hospital LOS**

| **Table S4** Unadjusted Odds Ratio’s for the association between ED LOS and hospital LOS of 3 or more days in the entire adult cohort | | | |
| --- | --- | --- | --- |
| **ED LOS** | **OR** | **95% CI** | **Number of patients** |
| **<4 hours** | 1.00 |  | 165,206 (94,516) |
| **4-8 hours** | 1.60 | 1.57 – 1.63 | 82,984 56,549() |
| **8-12 hours** | 1.83 | 1.72 – 1.93 | 5,697 (4,041) |
| **12-16 hours** | 2.14 | 1.87 – 2.46 | 1,082 (802) |
| **>16 hours** | 2.61 | 2.14 – 3.17 | 597 (464) |
| ^a^ Reference category in the regression analyses.  Patients with a hospital LOS of 3 or more days are shown between brackets for every subgroup.  Abbreviations: CI, confidence interval; ED, emergency department; LOS, length of stay; OR, odds ratio. | | | |

**Adjusted model hospital LOS**

| **Table S5** Adjusted Odds Ratio’s for the association between ED LOS and hospital LOS of 3 or more days in the entire cohort | | | |
| --- | --- | --- | --- |
| **ED LOS** | **OR** | **95% CI** | **Number of patients** |
| **<4 hours** | 1.00 |  | 164,206 (94,516) |
| **4-8 hours** | 1.40 | 1.38 – 1.43 | 82,984 (56,549) |
| **8-12 hours** | 1.56 | 1.47 – 1.66 | 5,697 (4,041) |
| **12-16 hours** | 1.82 | 1.58 – 2.10 | 1,082 (802) |
| **>16 hours** | 2.33 | 1.90 – 2.85 | 597 (464) |
| ^a^ Reference category in the regression analyses.  Patients with a hospital LOS of 3 or more days are shown between brackets for every subgroup.  In the final model we adjusted for the following confounders: age, triage category, top 10 presenting complaint, number of consultations in the ED, GCS, laboratory tests, radiological tests and discharge destination.  Abbreviations: CI, confidence interval; ED, emergency department; LOS, length of stay; OR, odds ratio. | | | |

**SUPPLEMENTAL FILE 7: Bradford Hill criteria**

The Bradford Hill criteria, proposed by Sir Austin Bradford Hill in 1965, offer a framework for determining causation from observed associations. These criteria encompass nine viewpoints: strength, consistency, specificity, temporality, biological gradient, plausibility, coherence, experiment, and analogy. While not a strict checklist, these viewpoints guide the evaluation process, emphasizing the need for comprehensive analysis before confirming a causal relationship^26^.

We assessed our study based on the Bradford Hill criteria, assigning scores to each criterion. A score of 0 indicates that the relationship between ED LOS and outcome measures does not fulfill the criterion of causation, 0.5 suggests a partial fulfillment, and a score of 1 indicates full fulfillment of the criterion.

**1. Strength of association (score: 1)**

Strength of association refers to the degree of increase in disease incidence associated with a particular factor. For example, in studying lung cancer among smokers, the significant increase in disease rates highlights the importance of this criterion. However, it's crucial to note that a weak association doesn't necessarily disprove a cause-and-effect hypothesis^26^.

In our study, the adjusted odds ratios across varying ED LOS categories (Figure 2 in main text) demonstrate a substantial connection between prolonged ED LOS and adverse outcomes, fulfilling this criterion.

**2. Consistency (score: 1)**

Consistency emphasizes the need for an observed association to be repeatedly confirmed by different researchers, in various places, circumstances, and times. This criterion ensures that the relationship between variables is not an isolated finding but holds true across diverse settings, strengthening the evidence for causation^26^.

The findings of the current study are consistent with six previous studies in health care systems with significantly longer ED LOS (see main text for articles). However, there were three conflicting findings, with some studies unable to show a significant association between ED LOS. However, we were able to explain this by variations in methodology, thus meeting the criteria consistency.

**3. Specificity (score: 0.5)**

This criterion evaluates whether the association is confined to specific groups or circumstances without plausible explanations other than the involvement of the studied factor. However, it’s crucial not to overemphasize this characteristic, as outcomes can have multiple causes^26^.

While our study highlighted a significant correlation between prolonged ED LOS and adverse outcomes, particularly affecting younger, non-urgently triaged patients, it's important to note that the observed association might not be entirely specific. Our discussion suggests that factors such as undertriage of non-urgent patients, the baseline hospital LOS of older urgently triaged patients, and other variables could contribute to the outcomes we observed. Therefore, our study partially meets this criterion. However, as Hill pointed out, this nuanced situation is not a drawback; rather, it underscores the existence of multiple potential causes at play, aligning with the complex reality of healthcare environments.

**4. Temporality (score: 1)**

Temporality involves establishing the sequence of events, ensuring that the exposure or factor being studied precedes the observed outcome. This chronological order is fundamental in demonstrating a cause-and-effect relationship^26^.

In our research, prolonged ED LOS preceded adverse outcomes, thus establishing a clear temporal relationship.

**5. Biological gradient (score: 1)**

This criterion examines whether the association displays a biological gradient or dose-response curve. For instance, the linear increase in lung cancer death rates with the number of cigarettes smoked daily provides strong evidence. A clear dose-response curve simplifies the relationship and strengthens the cause-and-effect hypothesis^26^.

In our study, we found a dose-response relationship for prolonged ED LOS in all of the subgroups (Figure 2 in the main text).

**6. Plausibility (score: 0.5)**

This criterion assesses whether the suspected causation aligns with biological understanding. However, demanding strict biological plausibility is not always feasible, as it depends on existing scientific knowledge. Unexplained associations should not be dismissed hastily; instead, they might signal new scientific discoveries. As Sherlock Holmes said, when all impossible explanations are eliminated, whatever remains, no matter how unlikely, must be considered^26^.

While it’s challenging to demand strict biological plausibility, our study carefully explores the association between ED LOS and adverse outcomes, considering factors such as age, triage urgency and disease severity. Although our study does not directly delve into the biological mechanisms, our findings align with existing biological knowledge concerning ED patients and their response to extended waiting times. An intriguing revelation from our study is the significant impact of prolonged ED stays on younger patients, particularly those categorized as non-urgent.

**7. Coherence (score: 1)**

The coherence criterion demands that the conclusions drawn from the data align with established knowledge about the disease or exposure without significant contradictions^26^.

Our findings cohere with established knowledge regarding ED operations and patient outcomes. They align with existing literature on the impact of extended waiting times in emergency settings. Furthermore, our study addresses any potential discrepancies or contradictions between our results and existing knowledge, providing explanations rooted in the specific context of our research.

**8. Experiment (score: 0.5)**

The experiment criterion assesses the impact of preventive actions taken based on observed associations. It examines whether these interventions effectively reduce the frequency of the associated events, providing strong support for the causation hypothesis if successful^26^.

In the context of ED LOS, a relevant study explored the impact of time-based targets on patient outcomes. This research aimed to determine whether interventions designed to shorten ED LOS led to tangible improvements. The findings, derived from multiple studies conducted across different countries, presented nuanced outcomes.
When considering hospital LOS, the studies offered diverse insights. New Zealand research suggested a moderate improvement, with fewer patients experiencing extended hospital stays after the introduction of time-based targets. In contrast, findings from Ireland did not demonstrate a significant association, underscoring the need for careful consideration of contextual factors. Additionally, Australian studies presented conflicting results; while some indicated longer hospital stays, others did not establish a significant impact.

In conclusion, existing studies on ED LOS interventions show mixed results. Some studies suggest a reduction in mortality and hospital LOS, while others show no significant impact. Thus, a definitive causal link between these interventions and patient outcomes remains uncertain. Further research is needed for a comprehensive understanding.

**9. Analogy (score: 1)**

The analogy criterion allows us to draw conclusions based on similar situations. For instance, if we have observed harmful effects of certain drugs or diseases during pregnancy, we might consider similar evidence for another drug or disease in pregnancy, even if the effects are less severe. It involves making comparisons to understand potential causal links^26^.

Our study indirectly draws on analogies from healthcare systems with longer ED LOS, reinforcing the idea that prolonged stays impact patient outcomes even in shorter ED LOS settings.

**SUPPLEMENTAL FILE 8: Potentially avoidable costs associated with prolonged hospital LOS**

**Number of patients per subgroup and discharge destination**

|  |  | **<4 hours** | **4-8 hours** | **>8 hours** | **Total** |
| --- | --- | --- | --- | --- | --- |
| **<70 non-urgent** |  |  |  |  | **17,044** |
|  | Ward | 11,305 | 5,039 | 266 | 16,610 |
|  | CCU/MCU | 224 | 87 | 8 | 319 |
|  | ICU | 61 | 53 | 1 | 115 |
| **<70 (very) urgent** |  |  |  |  | **117,049** |
|  | Ward | 65,966 | 34,537 | 3,048 | 103,551 |
|  | CCU/MCU | 5,314 | 1,191 | 118 | 6,623 |
|  | ICU | 5,599 | 1,169 | 107 | 6,875 |
| **≥70 non-urgent** |  |  |  |  | **15,331** |
|  | Ward | 9,371 | 5,396 | 247 | 15,014 |
|  | CCU/MCU | 184 | 75 | 6 | 265 |
|  | ICU | 27 | 21 | 4 | 52 |
| **≥70 (very) urgent** |  |  |  |  | **104,554** |
|  | Ward | 58,315 | 33,657 | 3,464 | 95,436 |
|  | CCU/MCU | 4,685 | 1,073 | 94 | 5,5852 |
|  | ICU | 2,488 | 707 | 71 | 3,266 |

**Calculations
Per subgroup**<70 non-urgent
Among the group of patients under the age of 70 with non-urgent triage categories (n=17,044), those who spent less than 4 hours in the ED had a predicted probability of 43.66% for a hospital stay longer than the median of 72 hours. For ED LOS between 4-8 hours, this probability increased to 58.08%, and for stays longer than 8 hours, it further rose to 62.40%. The 43.66% serves as a baseline probability for a hospital LOS exceeding 72 hours. This indicates that in the 4-8 hour group, there is a potentially preventable probability of prolonged hospital LOS of 14.42% (58.08% - 43.66%). In the group with ED LOS longer than 8 hours, this potential preventability increases to 18.74% (62.40% - 43.66%). Applying these percentage to the number of patients per subgroup and discharge destinations, we find that 777 (0.1442*5,039 + 0.1874*266), 14 (0.1442*87 + 0.1874*8), and 8 (0.1442*54 + 0.1874*2) patients could potentially spend less time in respectively the ward, CCU/MCU and ICU if the ED LOS had been shorter than 4 hours.

<70 (very) urgent
In this group, the baseline probability of hospital LOS exceeding the median was predicted to be 50.92%. For patients who spent between 4 and 8 hours in the ED, this prediction increased to 61.70% and for patients with ED LOS longer than 8 hours, it rose to 64.76%. This indicates a potential preventable prolonged hospital LOS of 10.78% in the 4-8 ED LOS group and 13.84% in the group with ED LOS longer than 8 hours, translating to 4,144 ward patients, 145 CCU/MCU patients, and 141 ICU patients.

≥70 non-urgent
Within this group, the baseline risk of a prolonged hospital LOS was predicted to be 63.60%. For patients with an ED LOS of 4-8 hours, this prediction increased to 73.44%, and for stays longer than 8 hours, it further rose to 75.70%. This indicates a potentially preventable prolonged hospital LOS of 9.84% in the 4-8 hour ED LOS group, and 12.1% in the group with ED LOS longer than 8 hours, translating to 561 ward patients, 8 CCU/MCU patients, and 3 ICU patients.

≥ 70 (very) urgent
Within this group, the baseline risk of a prolonged hospital LOS was predicted to be 66.56%. Among patients in this group, those with an ED LOS of 4-8 hours had a predicted probability of 75.51%, while patients with an ED LOS longer than 8 hours had a predicted probability of 78.37%. This indicates a potentially avoidable prolonged hospital LOS of 8.95% in the 4-8 hour group, and 11.81% in the group with ED LOS longer than 8 hours, translating to 3,421 ward patients, 107 CCU/MCU patients, and 72 ICU patients.

**Total cohort**In the total cohort, the unrounded number of patients with a potentially preventable prolonged hospital LOS adds up to 8,903 ward patients, 274 CCU/MCU patients and 224 ICU patients.

The entire cohort consisted of 623,861 patients. Therefore, the percentage of potentially preventable prolonged hospital LOS is approximately 1.43% (8,903 * 100 / 623,861) in patients who are admitted to the general ward and 0.04% in patients who are admitted to the CCU/MCU and ICU.

The average hospital LOS in the cohort of patients hospitalized for less than 3 days is 1.254 days in the regular ward, 0.915 days in the CCU/MCU and 1.097 days in the ICU. For patients hospitalized for 3 days or longer, the durations increased to respectively 8.402, 8.650, and 13.565 days. The difference between these groups is therefore respectively 7.148, 7.735, and 12.468 days.

**Total Dutch population**In the Netherlands, approximately 2,000,000 patients visit the ED annually. Using the unrounded versions of 1.43%, and 0.04%, this translates to 28,542 ward, 878 CCU/MCU, and 718 ICU patients with a potentially preventable prolonged hospital LOS per year. These patients contribute to respectively 204,018 (28,542*7.148), 6,791, and 8,952 additional hospital bed days per year.

In the Netherlands, the costs of a hospital ward bed are €700 per day, €1,307 per CCU/MCU and €2,224 per ICU day. Therefore, the potential preventable annual costs are €172,904,685 (204,018*700 +6,791 *1307 + 8,952 *2,224)
